# Associated factors for birth-related post-traumatic stress symptoms using a birth-specific measurement: a cross-sectional study

**DOI:** 10.1186/s12884-025-08517-9

**Published:** 2025-11-25

**Authors:** Greta Stén, Anna Malmquist, Katri Nieminen, Hanna Grundström

**Affiliations:** 1 Department of Obstetrics and Gynaecology in Norrköping, and Department of Biomedical and Clinical Sciences, Linköping University, Linköping, Sweden; 2 Department of Behavioural Sciences and Learning, Linköping University, Linköping, Sweden; 3 Department of Health, Medicine and Caring Sciences, Linköping University, Linköping, Sweden

**Keywords:** Birth-related PTSD, Postpartum PTSD, Risk factors, City birth trauma scale, City BiTS

## Abstract

**Background:**

One third of all childbirths are experienced as traumatic, which is a risk for developing birth-related PTSD (BR-PTSD). Understanding factors that increase the level of BR-PTSD symptoms (BR-PTSS) is crucial for the development of adequate preventive strategies. Most previous research has utilised general PTSD measurements, which negatively impacts its validity. This study therefore aimed to assess associated factors for BR-PTSS using a birth-specific instrument.

**Methods:**

In this cross-sectional study, BR-PTSS was measured using City Birth Trauma Scale. Information on prenatal and birth-related associated factors and comorbid symptoms of postpartum depression was collected via a self-report questionnaire including Childbirth Experience Questionnaire 2. Independent significant associated factors were analysed using single and multiple linear regression. The results were controlled for comorbidity with postpartum depression.

**Results:**

Independent significant associated factors were previous traumatic experience, primiparity, complications in pregnancy or childbirth, and a negative subjective experience of childbirth. When controlling for comorbidity with postpartum depression, significant associated factors were primiparity, complications in pregnancy or childbirth and a negative subjective experience of childbirth.

**Conclusions:**

This study identifies a negative subjective experience of birth as the most important associated factor for BR-PTSS and highlights the importance of modifiable factors. Our findings indicate that implementation of care acknowledging past trauma and primiparity, and evaluating the birth experience can help identify individuals with higher BR-PTSS. Future research should explore the effect on BR-PTSS when evaluating strategies focused on preventing and mitigating the experience of complications in pregnancy and birth, providing safe and supportive care, and promoting agency and self-efficacy during labour.

**Supplementary Information:**

The online version contains supplementary material available at 10.1186/s12884-025-08517-9.

## Introduction

Around 140 million births occur each year [1]. Of these, a noteworthy 34–45% are perceived as traumatic [2, 3]. Traumatic childbirth has been defined as an experience related to childbirth causing profound overwhelming/distressing emotions and reactions leading to a negative impact on the individual’s well-being [4]. Previously viewed as a subject of controversy, it is now acknowledged that traumatic childbirth may lead to post-traumatic stress symptoms (PTSS). Within the first period after the trauma, these symptoms are referred to as acute stress disorder [5]. While symptoms usually decrease within the first month post-partum, some individuals develop birth-related post-traumatic stress disorder (BR-PTSD). Adding to the substantial burden and distress of general PTSD, specific expressions and consequences of BR-PTSD include great fear of future childbirth, avoidance of future pregnancies [6], lower rates of breastfeeding, a negative impact on the child’s socio-emotional development [7], lower relationship satisfaction [8] and a potentially negative impact on the mental health of partners [9]. Research and awareness of BR-PTSD have increased in the past two decades. Nevertheless, significant knowledge gaps persist and lack of awareness among the public, policymakers and healthcare providers still exists [9–11].

According to the most recent Diagnostic Statistical Manual of Mental Disorders (DSM-5), PTSD is classified as a trauma and stress-related condition [5]. In BR-PTSD, the trauma criterion (criterion A) reflects on an event during, or near childbirth. Seven more criteria (B-H) must be fulfilled to classify for diagnosis. Symptoms are divided into four clusters: [1] re-experiencing symptoms (e.g., flashbacks, nightmares) [2], physical and mental avoidance [3], negative changes in cognition and mood (e.g., difficulty concentrating, fear, depressive symptoms,) and lastly [4] hyperarousal (hypervigilance, easily becoming scared or angry). The presence of a certain degree of PTSS not meeting all diagnostic criteria can be referred to as partial or subclinical PTSD [12]. As previous research has shown, individuals with partial PTSD can be affected negatively as well [7, 8, 13]. The fact that they do not classify for an established diagnosis may be a potential barrier to adequate care.

Results from meta-analyses show that BR-PTSD affects 3–5% of all people giving birth [9, 14–16]. Reviews have found a broad range of prevalence rates, which have been explained by differences in timing since childbirth, sample selection and data collection method [9, 17]. A broad range of questionnaires is being used [9], few of which address birth-related trauma or are up to date with DSM-5. The first self-report instrument based on DSM-5 and targeting BR-PTSD specifically (City Birth Trauma Scale, City BiTS) was developed in 2018, and facilitates reliable identification of BR-PTSD and measurement of birth-related PTSS (BR-PTSS) [18].

Trauma linked to BR-PTSD occurs in a unique context, differing from other types of traumatic events that might trigger PTSD. Childbirth is commonly expected and seen as a natural part of life, which is partly the reason why BR-PTSD has been a question of debate [9]. Furthermore, the peripartum period includes physical pain, hormonal alterations, breastfeeding, sleep deprivation and parent-baby bonding. Several theoretical models have been suggested for the understanding of PTSD but given the special birth context, in this work, we draw on the theoretical framework specific to BR-PTSD proposed by Ayers et al. [17]. Derived from the stress-diathesis model, this theoretical framework explains the development of PTSD as interactions between individual predisposing vulnerability factors (diatheses) and exposure to traumatic experiences (stressors). The vulnerability factors may impact the individuals’ resilience to traumatic experiences that, for some, occur in the peripartum period and increase the risk of developing PTSD. The model by Ayers et al. further divides the individual factors into pre-existing vulnerability factors, factors related to pregnancy or birth, and maintaining factors [17]. The stress sensitisation theory offers a framework for the mechanisms through which previous experiences and trauma may serve as a pre-existing vulnerability factor. This model suggests that exposure to psychological stress early in life sensitise the individual by long lasting alterations in neurobiological functions as well as emotional reactivity [19]. These changes may cause relatively low intense stressors to trigger severe psychological responses later in life. Knowledge of how different vulnerability factors and factors related to exposure of traumatic experiences are associated with BR-PTSS is important for developing adequate preventive strategies and peripartum care. Since birth-related trauma typically includes contact with healthcare professionals, better knowledge of modifiable factors could contribute to a reduction of symptoms and prevalence rates.

Key prenatal risk factors identified in previous research are depression during pregnancy, fear of childbirth, poor health and complications during pregnancy, and a history of PTSD. Further on, significant birth-related factors encompass subjective negative birth experiences, operative birth, inadequate support, and dissociation. Lastly, important postnatal risk factors identified are depression as well as stress and poor coping strategies [14, 15, 17]. Given that childbirth traditions and care vary globally, risk factors can be expected to differ between countries. Moreover, a recently published meta-analysis emphasises the importance of studies specifically identifying childbirth as the precipitating trauma to distinguish BR-PTSD/PTSS from PTSD/PTSS in the postnatal period resulting from traumatic experiences other than birth [9]. Hence, this study aimed to investigate associated factors for BR-PTSS using a birth-specific measurement.

## Methods

### Participants

This multicentre cross-sectional study with the aim to investigate associated factors for BR-PTSS using a birth-specific measurement was derived from the Swedish part of the International Survey of Childbirth-related Trauma (INTERSECT), an international project focusing on BR-PTSD [20]. Study participants were recruited during September 2021 to January 2022 from five maternity care clinics in different-sized cities in southern Sweden. Eligible participants were those aged *≥* 18 years, who had given birth 6–16 weeks prior to inclusion. Cases of stillbirth were excluded. Eligible study participants were identified via diagnostic codes in medical records. Mailing addresses were provided via the Swedish state personal address register. Sampling was done by stratified random sampling from those meeting the inclusion criteria. The study population was stratified by clinic, and thereafter randomised using Excel’s randomisation tool. The proportion of participants from each clinic was dependent on the size of the city. The original INTERSECT study recommended a sample size of 250, but since the response rate was expected to be low, a total of 2000 participants were contacted [20].

### Data collection

Data was collected via a self-report questionnaire. Information about the study and a link to an online version of the questionnaire was sent by mail. After two weeks, a reminder was sent to those who had not responded, together with a printed version of the questionnaire. The survey comprised questions regarding demographics, former and current mental health factors and aspects regarding the respondents’ most recent pregnancy and childbirth.

### Measurements

To assess BR-PTSS levels, the Swedish version of City BiTS (City BiTS-Swe) was used [21]. The instrument was developed to aid the diagnostic process of BR-PTSD while also assessing BR-PTSS levels [18]. It comprises 29 questions addressing all diagnostic criteria, symptom categories, and dissociative symptoms, and is answered with ‘yes’/’no’ or a four-graded Likert scale from 0 to 3, total range 0–60 points. Higher scores indicate a greater symptomatic burden.

To evaluate the subjective experience of childbirth, the Swedish Childbirth Experience Questionnaire 2 (CEQ2) was used [22]. The instrument consists of four subscales: professional support, agency, perceived security, and self-efficacy. Responses are on a four-point Likert scale [1–4], or a Visual Analogue Scale from 0 to 100 divided into four-scoring categories: 1 (0–40), 2 (41–60), 3 (61–80), and 4 (81–100). Negatively worded statements are reversed in score. The total score range is 1–4, a higher score indicating greater satisfaction and a more positive experience.

Postpartum depression symptoms were measured using the Swedish version of the Edinburgh Postnatal Depression Scale (EPDS) [23] originally developed by Cox, Holden and Sagovsky [24]. The instrument constitutes ten items, answered on a four-graded Likert scale (0–3). Total scores range from 0 to 30, higher values indicating more severe levels of depression symptoms.

### Data analysis

Selection of potential associated factors tested in this study were based on findings in previous studies on risk factors or associated factors for BR-PTSD/BR-PTSS, including the meta-analysis by Ayers et al. on which the theoretical framework is based [17]. Each factor was coded as 1 for the level presumed to positively correlate with the City BiTS score, while the other level was coded as 0. Categorical factors with more than two levels were dichotomised to enable linear regression analysis (Appendix A). Descriptive statistics were used for assessing demographical and birth-related aspects of the sample using mean, standard deviation, and percentage of valid answers. Univariable linear regression analyses were performed to identify significant associated factors for the total score of City BiTS. Factors with significant beta coefficients (*p* <.05) were then included in a multiple linear regression to detect independent associated factors using the forced entry method. Factors with fewer than ten observations at any level were excluded to minimise overfitting [25]. Since previous traumatic childbirth could only be assessed for multiparous individuals, it was excluded from the overall multiple regression to maintain statistical power. Separate regression analyses were then performed, stratified by parity, to assess the role of previous traumatic childbirth. Since comorbidity with BR-PTSS and postpartum depression symptoms is common, associated factors were controlled for EPDS scores in a second step. Multicollinearity was assessed with variance inflation factor (VIF), values below 5 indicating no considerable multicollinearity [26]. Assessment of model fit was done using adjusted R^2^, Fischer’s F-test and graphical assessment of residuals for the multiple regression models. Missing values were omitted pairwise in all analyses. The alpha level was set to < 0.05. All statistical analyses were conducted using the Statistical Package for Social Sciences Version 28.

## Results

Altogether, 619 individuals answered the survey, corresponding to a 31% response rate. The participants’ mean age was 32.8 (range 18–47), the majority stated that they lived in an urban environment, had a university education, an average or high household income in comparison to other households, identified as belonging to the ethnic majority and had been born in Sweden. Moreover, the majority were primiparous, had a term childbirth, had given birth vaginally, and did not experience any complications during pregnancy or childbirth. Full sample characteristics are shown in Table 1. A total of 610 individuals completed City BiTS with a total mean score of 7.9 ± 8.7 (range 0–55). The total mean score for CEQ2 was 3.3 ± 0.5 (range 1.3–4.0.3.0) and EPDS 6.2 ± 5.2 (range 0–26).

**Table 1:** Demographical, mental health-, pregnancy- and birth-related sample characteristics

		*n*	(%)	Mean (SD)
Age *(n* *= 619)*				32.7 (± 4.5)
Educational level *(n = 615)*	No education	1	(< 1)	
	Primary school	7	(1.2)	
	Upper secondary school	113	(18.1)	
	University	494	(79.2)	
Household income* *(n = 614)*	Below average	30	(4.8)	
	Average	336	(53.8)	
	Above average	248	(39.7)	
Marital status *(n* = *616)*	Married, registered partner	242	(38.8)	
	Partner, cohabitating	357	(57.2)	
	Partner, non-cohabitating	2	(< 1)	
	Single	10	(1.6)	
	Divorced	5	(1.0)	
Residential area *(n* = *617)*	Large city	361	(58.5)	
	Small city	178	(28.8)	
	Rural area	78	(12.6)	
Ethnicity *(n* = *587)*	Ethnic majority in Sweden	525	(84.1)	
	Ethnic minority in Sweden	62	(9.9)	
Country of birth *(n* = *616)*	Sweden	537	(86.1)	
	Other	79	(12.7)	
Previous traumatic experience** *(n* = *619)*	Yes	196	(31.7)	
	No	423	(68.3)	
Previous traumatic childbirth *(n = 335)*	Yes	128	(38.2)	
	No	207	(61.8)	
Parity *(n* = *619)*	Primipara	336	(53.8)	
	Multipara	283	(45.4)	
Multiple births *(n* = *619)*	Single	610	(98.5)	
	Twins	9	(1.5)	
Gestational age*** *(n* = *610)*				39.6 (± 1.8)
Mode of birth *(n = 619)*	Vaginal birth	458	(73.9)	
	Instrumental vaginal birth	48	(7.8)	
	Planned caesarean section	42	(6.8)	
	Emergency caesarean section	71	(11.5)	
Present birth partner *(n* = *619)*	Partner	599	(96.8)	
	Friend	5	(1.0)	
	Relative	8	(1.3)	
	Doula	1	(< 1)	
	None	6	(1.0)	
Complications in pregnancy/birth *(n* = *618)*	None	378	(60.6)	
	Minor	181	(29.0)	
	Major	59	(9.5)	
Complications, infant* (n* = *616)*	None	534	(85.6)	
	Minor	61	(9.8)	
	Major	21	(3.4)	

* % of valid answers *

*SD* standard deviations

*Household income in comparison to other households in Sweden

**Previous traumatic experience in accordance with DSM-5 criteria

***Week of delivery

Univariable regression analyses for detecting associated factors for BR-PTSS showed significant beta coefficients for three prenatal factors (previous traumatic experience, previous traumatic childbirth, parity) and five birth-related factors (prematurity, mode of birth, complications in pregnancy or birth, complications affecting the infant, subjective experience of childbirth). Moreover, the beta coefficient for postpartum depression was also significant. Two groups, those without birth partner and those giving birth to more than one child, comprised less than ten observations and additionally did not prove to be significant in the univariable analyses and were consequently left out of further analyses. Comprehensive results from univariable regression analyses for possible associated factors are shown in Table 2.

**Table 2:** Univariable linear regression for associated prenatal factors, birth-related factors, and comorbidity regarding symptoms of birth-related PTSD

	*n* (%) or M (± SD)		CityBiTS		Univariable linear regression			
			M (SD)		ß	95% CI	*R* ^2^	*p*
**Prenatal factors**								
Age *(n* = *619)*	32.8	(± 4.5)			− 0.135	− 0.289; 0.020	0.005	0.088
Educational level *(n* =* 615)*					− 0.483	−2.238; 1.272	0.000	*0.589*
University	494	(80.3)	8.0	(8.7)				
Other	121	(19.7)	7.5	(8.8)				
Household income (*n* = *614)*					− 0.401	−3.641; 2.840	0.000	*0.808*
Below average	30	(4.9)	7.5	(7.4)				
Other	584	(95.1)	7.9	(8.8)				
Living with partner *(n* = *616)*					−2.266	−6.520; 1.988	0.002	*0.296*
Yes	599	(97.2)	7.9	(8.8)				
No	17	(2.8)	5.7	(6.1)				
Ethnicity (*n* = *587)*					0.124	−2.200; 2.448	0.000	*0.917*
Swedish minority	62	(10.6)	8.1	(8.2)				
Swedish majority	525	(89.4)	8.0	(8.9)				
Country of birth *(n* = *616)*					0.202	−1.884; 2.288	0.000	*0.849*
Sweden	537	(87.2)	7.9	(8.6)				
Other	79	(12.8)	8.1	(9.8)				
Previous traumatic experience (*n* = *619)*					4.684	3.236; 6.133	0.062	***< 0.001***
Yes	196	(31.7)	8.1	(7.1)				
No	423	(68.3)	5.8	(7.4)				
Previous traumatic childbirth *(n* = *283)*					3.180	1.081; 5.279	0.031	***0.003***
Yes	108	(38.2)	7.6	(6.4)				
No	175	(61.8)	5.2	(6.7)				
Parity* (n* = *619)*					3.313	1.942; 4.685	0.036	***< 0.001***
Primipara	336	(54.3)	9.4	(9.9)				
Multipara	283	(45.7)	6.1	(6.7)				
**Birth-related factors**								
Multiple births (*n* = *619)*					5.177	− 0.660; 10.974	0.005	*0.071*
Yes	9	(1.5)	13.0	(13.0)				
No	610	(98.5)	7.9	(8.7)				
Prematurity (*n* = *610)*					4.355	0.966; 7.745	0.011	***0.012***
Yes	27	(4.4)	12.2	(12.1)				
No	586	(95.6)	7.7	(8.5)				
Mode of birth (*n* = *619)*					3.689	2.131; 5.247	0.034	***< 0.001***
Instrumental or operative birth	161	(26.0)	10.7	(11.2)				
Vaginal birth	458	(74.0)	7.0	(7.5)				
Present birth partner (*n* = *619)*					3.117	−3.980; 10.213	0.001	*0.389*
Yes	613	(99.0)	7.9	(8.6)				
No	6	(1.0)	11.0	(17.1)				
Complications in pregnancy/birth (*n* = *618)*					4.690	3.312; 6.069	0.068	***< 0.001***
Yes	240	(38.8)	10.8	(10.5)				
No	378	(61.2)	6.1	(6.8)				
Complications, infant (*n* = *616)*					4.333	2.309; 6.357	0.028	***< 0.001***
Yes	82	(13.3)	11.7	(10.3)				
No	534	(86.7)	7.4	(8.4)				
Subjective experience of birth (CEQ2) (*n* = *575)*	3.3	(± 0.5)			−9.201	−10.345; −8.057	0.306	***< 0.001***
**Comorbidity**								
Postpartum depression (EPDS) (*n* = *619)*	6.2	(± 5.2)			1.229	1.137; 1.322	0.529	***< 0.001***

Bold values indicate *p* <.05

*CEQ2* Childbirth Experience Questionnaire version 2,* EPDS* Edinburgh Postnatal Depression Scale

Significant independent associated factors found by multiple linear regression were previous traumatic experience, primiparity, complications in pregnancy or childbirth and a negative subjective experience of childbirth (Table 3). The model was statistically significant, F(7) = 45.042 *p* =.000, and explained 35% of the variance in BR-PTSS (adjusted R²=0.353). As shown in Table 3, the largest absolute standardised beta coefficient was found for subjective experience of birth (−0.497), followed by previous traumatic experience (0.148), and parity (0.124).

**Table 3:** Multiple linear regression for associated factors of symptoms of birth-related PTSD

	ß	95% CI	Std. ß	*p*
Previous traumatic experience	2.778	1.502; 4.055	0.148	***< 0.001***
Parity	2.168	0.969; 3.367	0.124	***< 0.001***
Prematurity	1.129	−1.816; 4.073	0.027	*0.452*
Mode of birth	− 0.350	−1.806; 1.106	− 0.018	*0.637*
Complications in pregnancy/birth	1.444	0.138; 2.750	0.081	***0.030***
Complications, infant	0.743	−1.115; 2.602	0.029	*0.432*
Subjective experience of birth (CEQ2)	−8.257	−9.451; −7.062	− 0.497	***< 0.001***

Bold values indicate *p* <.05. VIF was < 2 for all factors

*CEQ2 *Childbirth Experience Questionnaire version 2

Univariable and subsequent multiple regression analyses stratified by parity to assess the effect of previous traumatic childbirth in the multiparous group revealed that previous traumatic childbirth was significant in the univariable regression, but did not emerge as significant in the multiple regression analysis (1.215, 95% CI [−0.219; 2.650], *p* =.096) when taking into account other previous traumatic experience, complications in pregnancy or birth, complications affecting the infant and subjective birth experience (Appendix B).

When controlling for comorbidity with symptoms of postpartum depression, significant independent associated factors were parity, complications in pregnancy or childbirth and the subjective experience of childbirth (Table 4). Previous traumatic experience no longer reached a significant statistical level (0.943, *p* =.06). The model was statistically significant, F (8) = 121.313 *p* =.000, and explained 63% of the variance in BR-PTSS (adjusted R²=0.630). The largest absolute standardised beta coefficient was identified in postpartum depression (0.583), followed by the subjective experience of birth (−0.283) and parity (0.092).

**Table 4:** Multiple linear regression for associated factors of symptoms of birth-related PTSD controlled for postpartum depression comorbidity

	ß	95% CI	Std. ß	*p*
Previous traumatic experience	0.943	-0.038; 1.924	0.050	*0.060*
Parity	1.615	0.707; 2.523	0.092	***<0.001***
Prematurity	0.651	-1.576; 2.879	0.015	*0.566*
Mode of birth	0.591	-0.514; 1.696	0.030	*0.294*
Complications in pregnancy/birth	0.147	0.108; 2.085	0.061	***0.030***
Complications, infant	0.147	-1.259; 1.554	0.006	*0.837*
Subjective experience of birth (CEQ2)	-4.710	-5.675; -3.744	-0.283	***<0.001***
Postpartum depression (EPDS)	0.986	0.891; 1.081	0.583	***<0.001***

Bold values indicate* p*<.05. VIF was <2 for all factors

*CEQ2* Childbirth Experience Questionnaire version 2, *EPDS* Edinburgh Postnatal Depression Scale

## Discussion

In this study, we used multiple linear regression to investigate significant independent associated factors for birth-related post-traumatic stress symptoms (BR-PTSS) in a Swedish sample using a birth-specific PTSD measurement, the City Birth Trauma Scale (City BiTS). The sample presented overall low levels of BR-PTSS, a positive birth experience and low levels of postpartum depression symptoms, though some individuals reported severe symptoms and negative experiences. The mean score of City BiTS was similar to those in Spanish [27] and Hebrew-speaking samples [28], but lower than other European and Asian populations [18, 29–33].

The study found that previous traumatic experience, as defined in DSM-5, and first-time childbirth (primiparity) were significant prenatal vulnerability factors associated with increased BR-PTSS. Additionally, perceived complications in pregnancy or birth, and a negative subjective birth experience, were significant associated birth-related factors of BR-PTSS. These findings align with a previous study that also used City BiTS as a measurement [34], as well as research using general PTSD instruments [15, 17]. Moreover, a negative birth experience, assessed with CEQ2, emerged as the most important associated factor. The mean score of CEQ2 (3.3 ± 0.5) was similar to a large Swedish community sample (3.4 ± 0.4) [35], but higher than findings in Iranian (2.7 ± 0.7) [36], Finnish (2.9 ± 0.5) [37] and Portuguese (2.9 ± 0.5) [38] samples. Although taking into account that birth experience was measured at the same time as BR-PTSS, previous research including several meta-analyses has consistently linked birth experience with BR-PTSD using a variety of measures [14, 15, 17, 39]. It is therefore plausible that methods aiming to improve the experience of childbirth might lead to a decrease in BR-PTSS. For example, continuous support during labour has been linked to a lower risk for a negative birth experience [40]. In addition, CEQ2 covers several aspects of the birth experience: professional support, agency, perceived security, and self-efficacy. Since these are all modifiable factors, high-quality care targeting mentioned fields is also likely to lead to improved mental health postpartum. As exemplified by a previous study, one way to enhance agency and self-efficacy is to involve the birth-giver in the decision-making regarding interventions during birth since this seems to promote a sense of personal achievement and control [41].

Perceived complications in pregnancy or birth, also found to be an important associated factor for BR-PTSS, is also a modifiable factor to a certain degree. Conversely, instrumental or operative birth, highlighted as a risk factor in a previous meta-analysis [17] did not reach a significant level in the multiple regression, indicating that the subjective perception is more important than the medical complication itself in the development of BR-PTSS. While not all complications can be prevented, medical staff can partially reduce the risk and affect how the individual perceives the medical complication or intervention. Approaches that prevent and mitigate complications may therefore decrease BR-PTSS, as exemplified by a systematic review suggesting that preparedness for complications and strategies to minimise interventions improve the birth experience [42]. In contrast, primiparity and previous traumatic experience, both emerging as important associated factors in this study, cannot be modified within perinatal care in a similar way. Knowledge of their association with BR-PTSD among caregivers is nonetheless important to ensure the best possible support. Our findings lend support to the stress sensitisation theory [19], suggesting that previous traumatic experiences have a negative impact on the psychological reaction following exposure to traumatic events during childbirth later in life. The association between primiparity and elevated BR-PTSS may, in part, be explained by a lower level of psychological preparedness for childbirth compared to multiparous individuals. However, it might also reflect a higher prevalence of fear of childbirth among primiparous women—a pattern observed in previous research [17, 43], though not examined in the present study due to its cross-sectional design. Tailored preventive strategies and peripartum care protocols may be relevant for both primiparous and previously traumatised individuals. Overall, the significant associated factors identified in this study support the view advocated by Horsch et al. [11] that low-cost healthcare improvements of recognition of past trauma, providing good support and communication during labour, and assessing childbirth experience could improve BR-PTSD care. Similarly, interventions providing trauma-informed support such as psychological first aid administered shortly after the trauma, in this case the childbirth, may present another relatively low-cost method to further tailor postnatal care to individuals at elevated risk of developing BR-PTSS. This approach has shown promising results in both in birth-related context [44] and in PTSD stemming from other types of trauma [45].

Our results indicate substantial comorbidity between postpartum depression and BR-PTSS, consistent with meta-analyses and a systematic review based on studies using general PTSD measurements [14, 15, 17]. When controlling for comorbid symptoms of postpartum depression, the most important associated factors for BR-PTSS were primiparity, complications in pregnancy or birth, and a negative subjective experience. Since comorbidity between the two conditions is common, it is important to note that the above-mentioned factors are associated with BR-PTSS both in those with and without comorbid depression. Even though symptoms partly overlap, recommended treatment and supportive interventions are not identical [11, 46]. Although postpartum depression awareness is more widespread, screening protocols peripartum regularly overlook BR-PTSD [47]. Therefore, our study highlights the importance of considering BR-PTSD as a potential differential diagnosis or comorbidity in individuals with symptoms of postpartum depression in combination with a negative subjective birth experience, pregnancy or birth complications, as well as in primiparous individuals.

Previous traumatic childbirth did not prove to be an associated factor for BR-PTSS in the multiparous group. This is interesting since previous traumatic event was shown to be a significant associated factor. This implies that other types of traumatic events might play a more important role than previous traumatic childbirth in the development of BR-PTSS. However, the result is possibly due to underpowered analysis since the multiparous group was much smaller (*n* = 283) in comparison to the total sample (*n* = 610).

None of the examined demographical factors were significantly associated with BR-PTSS. The lack of significant results may be attributed to small participant numbers in certain groups, such as foreign-born individuals and lower educational level. As suggested in a systematic review, socioeconomic rank probably has a limited impact on BR-PTSD development since findings have been inconsistent in previous studies [48]. Hence, the effect sizes of most demographic factors are likely to be small despite a larger sample size.

This is one of few studies addressing associated factors using a birth-specific measurement, which enhances the validity of the results [34]. Another strength is the investigation of associated factors for PTSS rather than full PTSD diagnosis, increasing applicability to individuals with partial or subclinical PTSD. Strengths of this study also include the use of a validated instrument for measuring childbirth experience complexity, and the use of DMS-5 protocols for previous trauma assessment. The sample aligns with Swedish birth-giving individuals regarding key demographics (age, rates of multiple births, premature birth, emergency and planned caesarean sections, instrumental vaginal births) [49, 50], making the sample representative of the population overall. Readers should nevertheless take note of certain limitations. The use of self-reporting introduces a certain degree of self-report and nonresponse bias. The latter is evident from some of the sample characteristics, where the proportion of primiparous and highly educated individuals was higher than the corresponding national average, and the proportion of foreign-born persons was considerably lower [49–51] potentially impacting the significance of demographic factors in this study. Furthermore, as most participants in this sample did not present with known risk factors, the results are generalisable to the broader population; however, additional studies focusing on specific subgroups at elevated risk would be valuable to determine whether their outcomes differ. Moreover, due to the cross-sectional design it is not possible to prove causal relationship between the associated factors and more severe BR-PTSS in this study. Similarly, it was not possible to assess the role of prenatal mental illness, fear of childbirth and postpartum depression as predictive factors, or the effect of postpartum support and coping strategies, due to the cross-sectional rationale.

Further studies using birth-specific instruments such as City BiTS for measurement would enable even more reliable meta-analysis of predictive factors for BR-PTSS. Research investigating factors associated with various symptomatic profiles, as conducted by Staudt et al., would broaden understanding of risk factors and comorbidity [52]. Moreover, exploring less studied factors such as sexual and gender minorities is important to ensure optimal care. Lastly, controlling for other comorbidities such as anxiety would provide additional insight into associated factors for individuals with multiple mental health concerns.

## Conclusions

To conclude, this study is one of few assessing associated factors for BR-PTSS specifically identifying childbirth as the precipitating trauma. A negative subjective birth experience was identified as the most important associated factor of BR-PTSS, and the study highlights the significance of modifiable factors and postpartum depression comorbidity. Our findings indicate that implementation of care acknowledging past trauma and primiparity, and evaluating the birth experience, can help identify individuals with higher BR-PTSS. Enhancing strategies to prevent or mitigate the experience of complications in pregnancy and birth, provide safe and supportive care, and promote agency and self-efficacy during labour, may reduce BR-PTSS, further explored in future research.

## Supplementary Information

Supplementary Material 1. Dichotomisation and coding for categorical factors with more than two levels and gestational age. Appendix A displays a table with the dichotomisation and coding for categorical factors with more than two levels and gestational age.

Supplementary Material 2. Multiple linear regression for associated factors of symptoms of birth-related PTSD in multiparous individuals, presented with beta coefficients with 95% confidence intervals. Appendix B displays a table presenting the results of multiple linear regression for associated factors of symptoms of birth-related PTSD in multiparous individuals.

## Data Availability

The datasets generated and analysed during the current study are not publicly available due to ethical reasons but are available from the corresponding author on reasonable request.

## Abbreviations

BR-PTSD: Birth related post-traumatic stress disorder
BR-PTSS: Birth related post-traumatic stress symptoms
CEQ2: Childbirth experience questionnaire 2
City BiTS: City birth trauma scale
City BiTS-Swe: City birth trauma scale swedish version
DSM-5: Diagnostic statistical manual of mental disorders 5
EPDS: Edinburgh postnatal depression scale
INTERSECT: International survey of childbirth-related trauma
PTSD: Post-traumatic stress disorder
PTSS: Post-traumatic stress symptoms
VIF: Variance inflation factor
