# Supplementary material for: Associated factors for birth-related post-traumatic stress symptoms using a birth-specific measurement: a cross-sectional study
Source: BMC Pregnancy Childbirth. 2025 Nov 25;25:1301. doi: 10.1186/s12884-025-08517-9 (PMC12690891; doi:10.1186/s12884-025-08517-9)
Supplement: Supplementary file 1 — Supplementary Material 1. Dichotomisation and coding for categorical factors with more than two levels and gestational age. Appendix A displays a table with the dichotomisation and coding for categorical factors with more than two levels and gestational age. [file 12884_2025_8517_MOESM1_ESM.pdf]

Appendix A. Dichotomisation and coding for categorical factors with more than two levels and gestational age.

Factor	Dichotomisation	
	Coded as 0	Coded as 1
Educational level	University education	No education<br>Primary school<br>Upper secondary school
Household income	Average<br>Above average	Below average
Marital status*	Married<br>Cohabitating partner	Single<br>Widow<br>Non-cohabitating partner<br>Divorced
Gestational age**	Delivery≥37 weeks	Delivery <37 weeks
Mode of birth	Vaginal birth	Instrumental vaginal birth<br>Planned caesarean section<br>Emergency caesarean section
Present birth partner	Partner<br>Friend<br>Relative<br>Doula	No present partner
Complications in pregnancy/birth	No complications	Minor complications<br>Major complications
Complications, infant	No complications	Minor complications<br>Major complications

*Note: *Marital status was converted to 'living with partner'. **Gestational age was converted to the categorical variable 'prematurity'.*
