# Supplementary material for: Associated factors for birth-related post-traumatic stress symptoms using a birth-specific measurement: a cross-sectional study
Source: BMC Pregnancy Childbirth. 2025 Nov 25;25:1301. doi: 10.1186/s12884-025-08517-9 (PMC12690891; doi:10.1186/s12884-025-08517-9)
Supplement: Supplementary file 2 — Supplementary Material 2. Multiple linear regression for associated factors of symptoms of birth-related PTSD in multiparous individuals, presented with beta coefficients with 95% confidence intervals. Appendix B displays a table presenting the results of multiple linear regression for associated factors of symptoms of birth-related PTSD in multiparous individuals. [file 12884_2025_8517_MOESM2_ESM.pdf]

Appendix B. Multiple linear regression for associated factors of symptoms of birth-related PTSD in multiparous individuals, presented with beta coefficients with 95% confidence intervals.

	ß	95% CI	*p*
Previous traumatic childbirth	1.215	-.219; 2.650	*.096*
Previous traumatic experience	3.194	1.628; 4.760	***<.001***
Complications in pregnancy/birth	.551	-1.001; 2.102	*.485*
Complications, infant	1.501	-1.052; 4.053	*.248*
Subjective experience of birth (CEQ2)	-5.847	-7.277; -4.417	***<.001***

Note: CEQ2 = Childbirth Experience Questionnaire version 2. Bold values indicate p<.05. VIF was <2 for all factors.
